# PineRipeSeg: a lightweight texture-edge-aware network for pineapple instance segmentation and ripeness classification in field environments

**DOI:** 10.3389/fpls.2026.1953890

**Published:** 2026-09-23

**Authors:** Hang Shi, Lin Wang, Nuo Li, Changxi Liu, Hao Sun, Hailiang Li, Jun Hu

**Affiliations:** 1 College of Engineering, Heilongjiang Bayi Agricultural University, Daqing, China; 2 South Subtropical Crops Research Institute, Chinese Academy of Tropical Agricultural Sciences, Key Laboratory of Tropical Fruit Biology, Ministry of Agriculture and Rural Affairs, Zhanjiang, China; 3 Heilongjiang Engineering Technology Research Center for Potato Mechanization, Daqing, China

**Keywords:** instance segmentation, lightweight network, pineapple, ripeness classification, texture-edge decoupling attention

## Abstract

**Introduction:**

Dense leaf occlusion and fruit–leaf feature confusion hinder simultaneous extraction of pineapple instance contours and appearance-based ripeness classification in field images.

**Methods:**

A dataset of 6,019 field images and 9,393 polygon-annotated Bali pineapple instances was constructed, with fruits categorized as Unripe, Partially ripe, or Ripe according to standardized external-appearance criteria. PineRipeSeg, a lightweight YOLO11n-seg-based network, was developed. Its principal contribution is the proposed Texture-Edge Decoupling Attention (TEDA) module, which exploits the contrast between multidirectional pineapple-eye textures and elongated leaf edges to enhance fruit-surface features and suppress leaf interference. GhostConv reduces redundant computation, whereas an intersection-over-union (IoU) loss with scaled auxiliary boxes, termed Inner-IoU, refines occluded-fruit localization.

**Results:**

Mean average precision (mAP) reached 98.1% for boxes at IoU 0.5, 98.0% for masks at IoU 0.5, and 82.2% for masks across IoU 0.5 -0.95, exceeding YOLO11n-seg by 5.0, 5.2, and 3.4 percentage points, respectively. Missed detections decreased by 77.8%. The model required 8.7 giga floating-point operations (GFLOPs), occupied 5.1 MB, and processed 121 frames per second (FPS). Comparative, ablation, repeated-run, challenging-scene, and heatmap analyses supported its effectiveness under occlusion and similarly colored backgrounds.

**Discussion:**

PineRipeSeg provides a lightweight two-dimensional method for occlusion-aware hybrid instance segmentation and external ripeness assessment in field environments.

## 1 Introduction

Pineapple (*Ananas comosus*) is an important commercial crop in the tropical and subtropical regions of southern China, with concentrated production in Guangdong, Hainan, and Guangxi (Li D. et al., 2022). Field harvesting still relies heavily on manual labor under hot conditions and among sharp leaves, making the work physically demanding. Automated or intelligent harvesting equipment could reduce dependence on manual labor and improve harvesting efficiency. Reliable information on fruit position, contour, and ripeness provides the visual basis for subsequent robotic localization and harvesting operations (He F. et al., 2024; Xiao et al., 2025).

Visual perception of pineapples under natural field conditions presents distinct crop-specific challenges. Pineapple fruits usually develop within a canopy of dense, sword-shaped leaves. These long, interlaced leaves cause occlusion at different positions and to varying degrees, leaving visible fruit regions incomplete, discontinuous, and irregularly bounded. Changes in natural illumination also alter the apparent surface color. Unripe fruit can resemble green leaves, whereas ripe fruit can resemble senescent yellow leaves. The crown and surrounding leaves both exhibit prominent linear edges and radial structures. A model may therefore focus on the crown or leaves while neglecting the interlocking texture of pineapple eyes and the color-transition regions that more directly characterize fruit identity and ripeness. Previous studies have likewise identified leaf occlusion, illumination variation, target overlap, and complex backgrounds as major factors affecting the reliability of field pineapple recognition (Chang et al., 2022; Lai et al., 2023; Meng et al., 2023).

In recent years, deep learning has been applied to pineapple detection, ripeness classification, and the extraction of visual information for harvesting. Chang et al. (2022) combined deep learning with distance measurement to identify pineapple ripeness and three-dimensional distance. Lai et al. (2023) detected Unripe, Partially ripe, and Ripe pineapples in the field using an improved YOLOv7 model, and used an attention mechanism and Soft-NMS to improve recognition under occlusion. Meng et al. (2023) developed a spatiotemporal convolutional neural network with Transformer-based feature modeling for pineapple detection under complex field conditions. Zhang R. et al. (2024) proposed MSGV-YOLOv7, which reduced computational complexity through a lightweight backbone and a feature-fusion structure. These studies demonstrate the feasibility of deep learning for field pineapple recognition and ripeness classification, but they mainly output rectangular bounding boxes. A bounding box provides an approximate fruit location but inevitably includes pixels from leaves, soil, and other background elements. It cannot accurately describe the boundary of the visible fruit surface under severe occlusion and does not readily exclude interference from the crown and sword-shaped leaves.

Compared with bounding-box detection, instance segmentation generates a pixel-level mask for each fruit, enabling individual targets to be separated from surrounding leaves and background regions more effectively. In the present study, the annotation target follows an occlusion-aware hybrid protocol: visible regions are retained when the hidden contour cannot be inferred reliably, whereas limited local contour completion is allowed when the occluded fruit boundary can be determined with sufficient confidence (Kang and Chen, 2020; Sheng et al., 2023; Wei et al., 2025; Shan et al., 2025). Kang and Chen (2020) used a multitask network to detect and segment orchard apples simultaneously. Sheng et al. (2023) introduced edge-guided and boundary-aware structures to improve fruit-edge segmentation under complex illumination, fruit overlap, and leaf occlusion. Wei et al. (2025) used instance segmentation for tomato contour extraction and ripeness recognition, showing that pixel-level masks can support both fruit-instance separation and appearance-state classification. For mechanized pineapple harvesting, Shan et al. (2025) proposed MAISNet to extract pineapple locations and geometric contours in real time, demonstrating the potential of instance segmentation for detailed pineapple perception.

Recent studies in related learning tasks have also explored complementary strategies for improving feature discrimination, model efficiency, and adaptation to heterogeneous data. In agricultural vision, PestScope introduced exclusion-aware reasoning to suppress non-target regions during fine-grained pest segmentation (Yang et al., 2026). Beyond agricultural imaging, WPMNet combined multi-scale feature extraction with weight pruning and parameter quantization to improve model efficiency (Wang D. et al., 2026), while Meta-TIP employed unsupervised style-adaptive learning to accommodate differences across multiple image datasets (Ma B. et al., 2025). These studies reflect broader efforts to improve task-specific representation, computational efficiency, and robustness under complex data conditions. Related studies have also explored context-aware multi-scale feature recovery under imaging degradation (Peng et al., 2026) and resource-aware collaborative inference under latency and energy constraints (Jin et al., 2026), further illustrating the broader development of robust representation and efficient intelligent inference.

However, existing pineapple studies have generally addressed either bounding-box-based ripeness detection or fruit-contour segmentation. Although recent fruit-segmentation methods have improved feature representation through attention mechanisms, multiscale fusion, or edge enhancement, these strategies generally lack explicit modeling of the crop-specific structural conflict in field pineapple images. Pineapple eyes form a multidirectional interlocking texture, whereas sword-shaped leaves and crowns are dominated by elongated linear edges. Under severe occlusion, generic spatial or edge enhancement may strengthen both structures, making it difficult to distinguish visible fruit-surface features from leaf interference. A lightweight framework that jointly predicts occlusion-aware hybrid instance masks and appearance-based ripeness classes while explicitly exploiting this texture–edge difference is still lacking.

To address these problems, the aim of this study was to develop a lightweight instance-segmentation model capable of simultaneously segmenting field pineapple instances under occlusion and classifying their appearance-based ripeness under severe leaf occlusion, fruit–leaf feature confusion, and variable natural illumination. Accordingly, PineRipeSeg was developed based on YOLO11n-seg. The model outputs the fruit class, bounding box, confidence score, and instance mask, thereby providing integrated visual information on pineapple identity, appearance-based ripeness, and annotated fruit instance contours.

The main contributions of this study are as follows:

1. A field pineapple instance-segmentation dataset with three appearance-based ripeness classes—Unripe, Partially ripe, and Ripe—was constructed. The dataset covers different years, plots, acquisition devices, viewing angles, illumination conditions, and degrees of occlusion. Fruit polygon masks follow an occlusion-aware hybrid annotation protocol that combines visible-region labeling with partial amodal completion when the hidden contour can be reliably inferred. Images were grouped by acquisition year and plot to reduce data leakage between closely related samples.
2. PineRipeSeg was developed as a lightweight instance-segmentation network based on YOLO11n-seg. Its principal methodological contribution is the proposed Texture-Edge Decoupling Attention (TEDA) module. TEDA exploits the structural difference between the multidirectional interlocking texture of pineapple eyes and the elongated linear edges of leaves and crowns. It strengthens spatial responses on visible fruit surfaces while reducing interference from non-fruit linear structures. A residual-scaling pathway retains the original feature stream during attention recalibration. GhostConv and Inner-IoU were further incorporated as complementary components to reduce redundant computation and improve bounding-box regression for occluded targets, respectively.
3. PineRipeSeg was evaluated through comparisons with representative instance-segmentation models, module-ablation experiments, repeated comparisons among attention mechanisms, challenging-scene analysis, missed-detection statistics, confusion-matrix analysis, and class-activation heatmaps. These experiments assessed its localization, occlusion-aware hybrid instance segmentation, appearance-based ripeness classification, and computational performance under natural field conditions.

## 2 Materials and methods

### 2.1 Field image acquisition

The field images used in this study were collected from pineapple-growing areas in Tiaofeng Town, Leizhou, and Jinhe Town, Xuwen County, Zhanjiang, Guangdong Province, China (**Figure 1**). Data collection was conducted in two stages: 18–21 April 2025 and 19–28 March 2026. The images depicted field-grown pineapples of the Bali cultivar at the Unripe, Partially ripe, and Ripe stages. Field conditions differed between locations and years in planting density, natural illumination, soil background, leaf growth, and degree of fruit occlusion. The dataset therefore included natural field samples with variation across locations and years.

**Figure 1:**
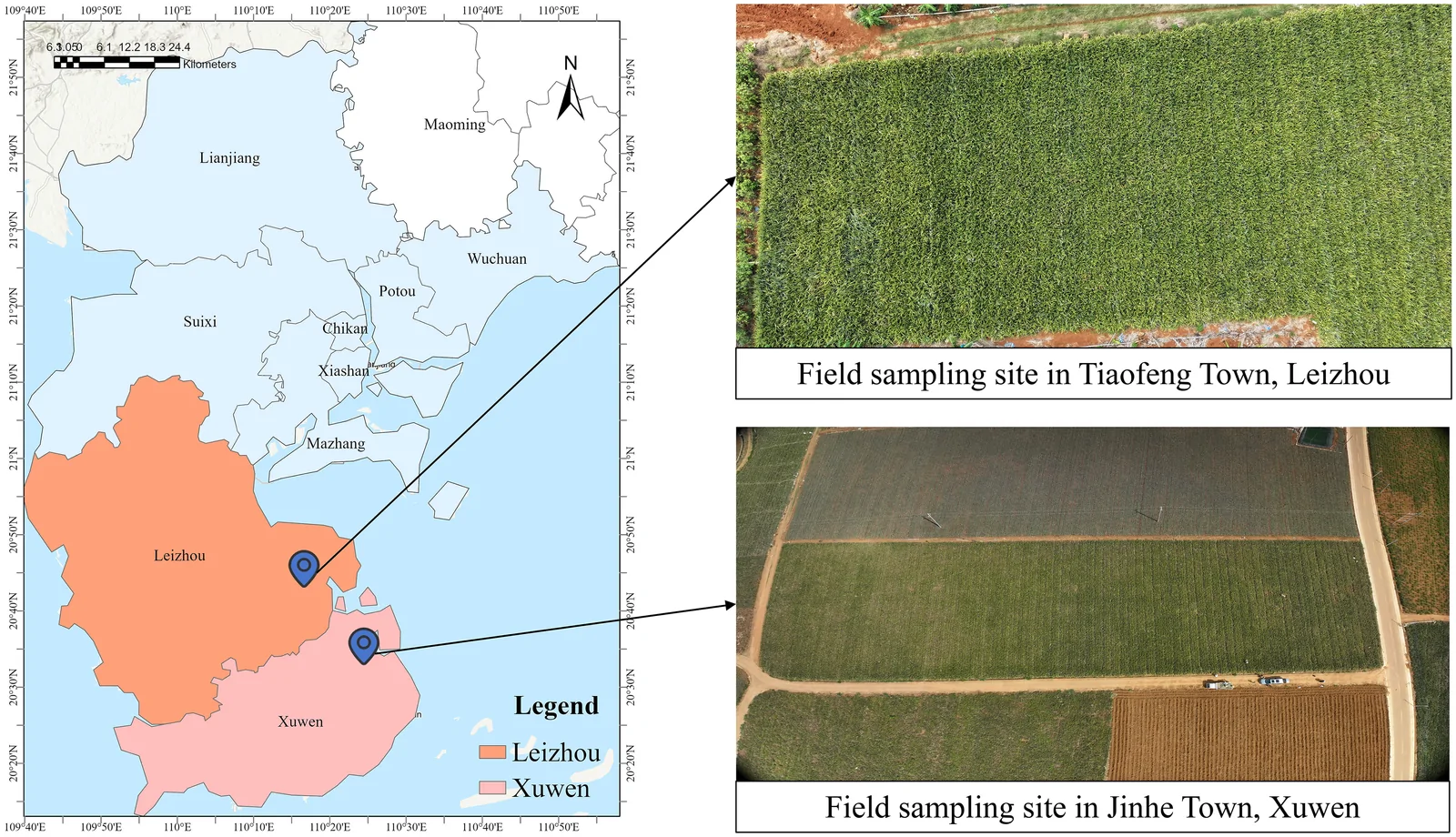
Image-acquisition locations.

To improve model generalization across the imaging characteristics of different optical sensors, a mixed, multi-device acquisition strategy was adopted. High-resolution field images were captured with a Hikvision industrial camera and Huawei nova 11 Pro, iPhone 13 Pro Max, and iPhone 16 Pro Max mobile devices. Core device specifications are provided in **Table 1**. The camera-to-fruit distance was maintained at approximately 1.0–1.5 m, and images were captured from frontal, lateral, and oblique views. After manual screening, 4850 valid original images were retained.

**Table 1:** Specifications of the image-acquisition devices.

Model	Resolution (pixels)	Images acquired
Hikvision DS-UVC-U64 PRO	2560×1440	1530
Huawei nova 11 Pro	3072×4096	1061
iPhone 13 Pro Max	3840×2160	967
iPhone 16 Pro Max	3840×2160	1292

The resulting field images contained pineapple samples at different ripeness stages, with varying degrees of leaf occlusion, fruit scales, viewing angles, and background complexity. Some ripe fruit closely resembled senescent yellow leaves in color, and only small portions of some fruit were exposed outside the canopy. These conditions increased the difficulty of extracting instance contours and classifying ripeness. Representative samples are shown in **Figure 2**, including partially occluded pineapple, severely occluded pineapple, partially decayed pineapple, and densely distributed multiple targets.

**Figure 2:**
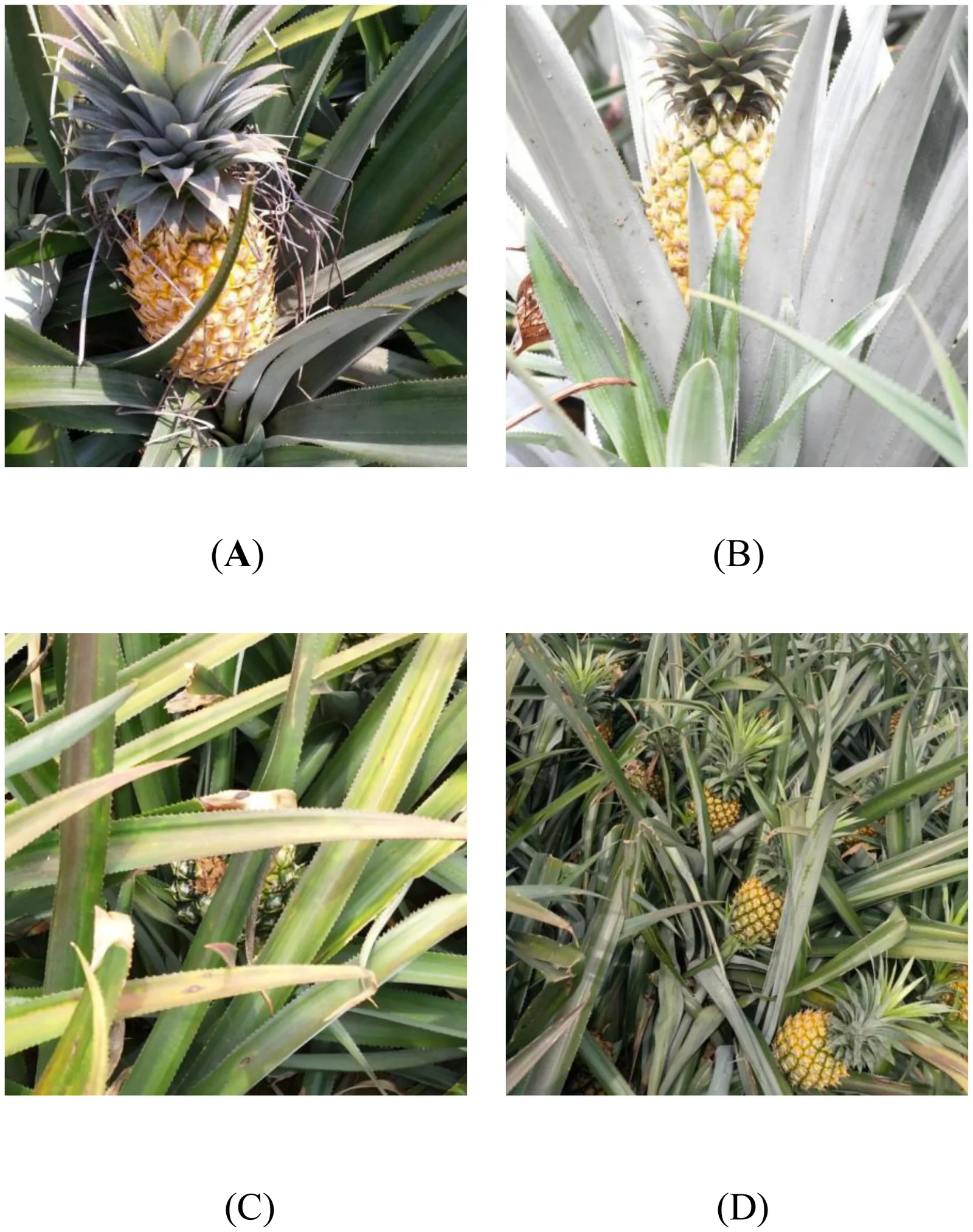
Representative pineapple images acquired in the field. **(A)** Partially occluded pineapple. **(B)** Severely occluded pineapple. **(C)** Partially decayed pineapple. **(D)** Densely distributed multiple targets.

### 2.2 Image augmentation

To increase the diversity of the pineapple-ripeness dataset, data augmentation was applied only to the training images after the training, validation, and test sets had been separated. Every geometric transformation simultaneously updated the instance mask, bounding box, and ripeness-class label to maintain consistency between each augmented image and its annotations. The augmentations included affine transformations, such as random cropping, horizontal flipping, translation, rotation, and scaling, as well as Gaussian noise, mild motion blur, Cutout, and Mosaic (Ji M. et al., 2024) (**Figure 3**). Cutout simulated partial fruit occlusion by leaves, whereas motion blur simulated imaging disturbance during harvesting movement. Because peel color is an important indicator of pineapple ripeness, transformations that could alter the true color distribution, including changes in hue or saturation, were not used. These methods increased the diversity of the training data while preserving the ripeness class and fruit-contour characteristics. After augmentation, the dataset contained 6019 images.

**Figure 3:**
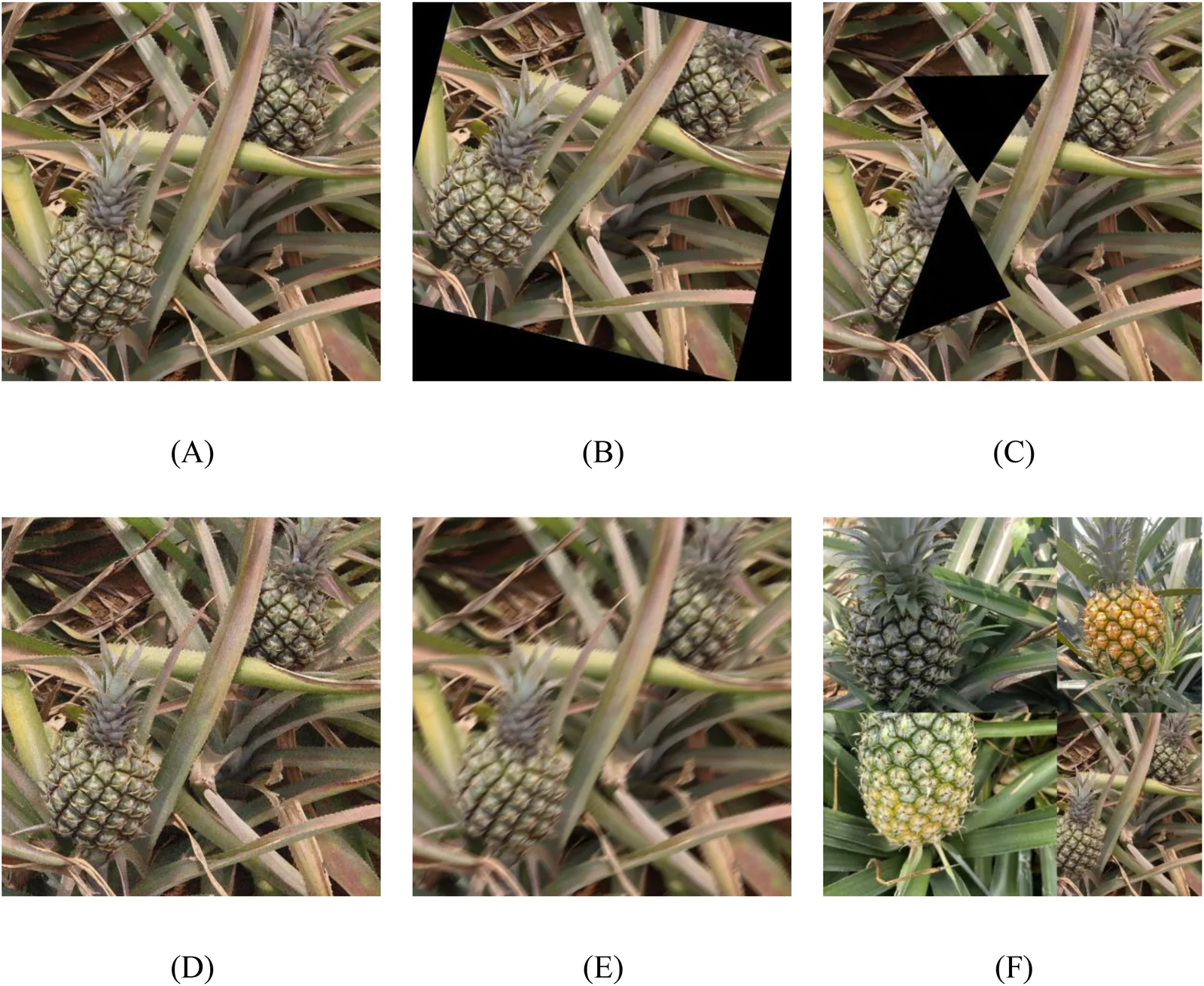
Image-augmentation methods. **(A)** Original image. **(B)** Affine transformation. **(C)** Cutout. **(D)** Gaussian noise. **(E)** Motion blur. **(F)** Mosaic.

### 2.3 Pineapple ripeness-grading criteria

Scientifically defined and consistent dataset labels are essential for reliable instance segmentation and ripeness classification. The appearance-based ripeness criteria specified in Appendix A of the Chinese agricultural industry standard NY/T 450-2001, Pineapple, were adopted. Based on the expansion of orange-yellow areas over the fruit surface, the standard divides naturally ripened summer fruit into four stages. At stage 1, the fruit is predominantly green and the grooves between fruitlets show slight yellowing. At stage 2, orange-yellow coloration begins at the fruit base. At stage 3, the orange-yellow region expands from approximately one-quarter to one-half of the lower fruit. At stage 4, this region expands from approximately half of the fruit to the entire surface.

Stages 2 and 3 both represent the continuous transition of the peel from green to orange-yellow. Under natural field conditions, their visual boundary is readily affected by illumination, leaf occlusion, and variation among individual fruit. Accordingly, stages 2 and 3 were combined into a Partially ripe class, resulting in three appearance-based ripeness classes: Unripe, Partially ripe, and Ripe. Standard stage 1 corresponded to Unripe, stages 2 and 3 corresponded to Partially ripe, and stage 4 corresponded to Ripe. Representative visual phenotypes and their corresponding polygon annotations are shown in **Figure 4**, and the quantitative grading criteria are listed in **Table 2**.

**Figure 4:**
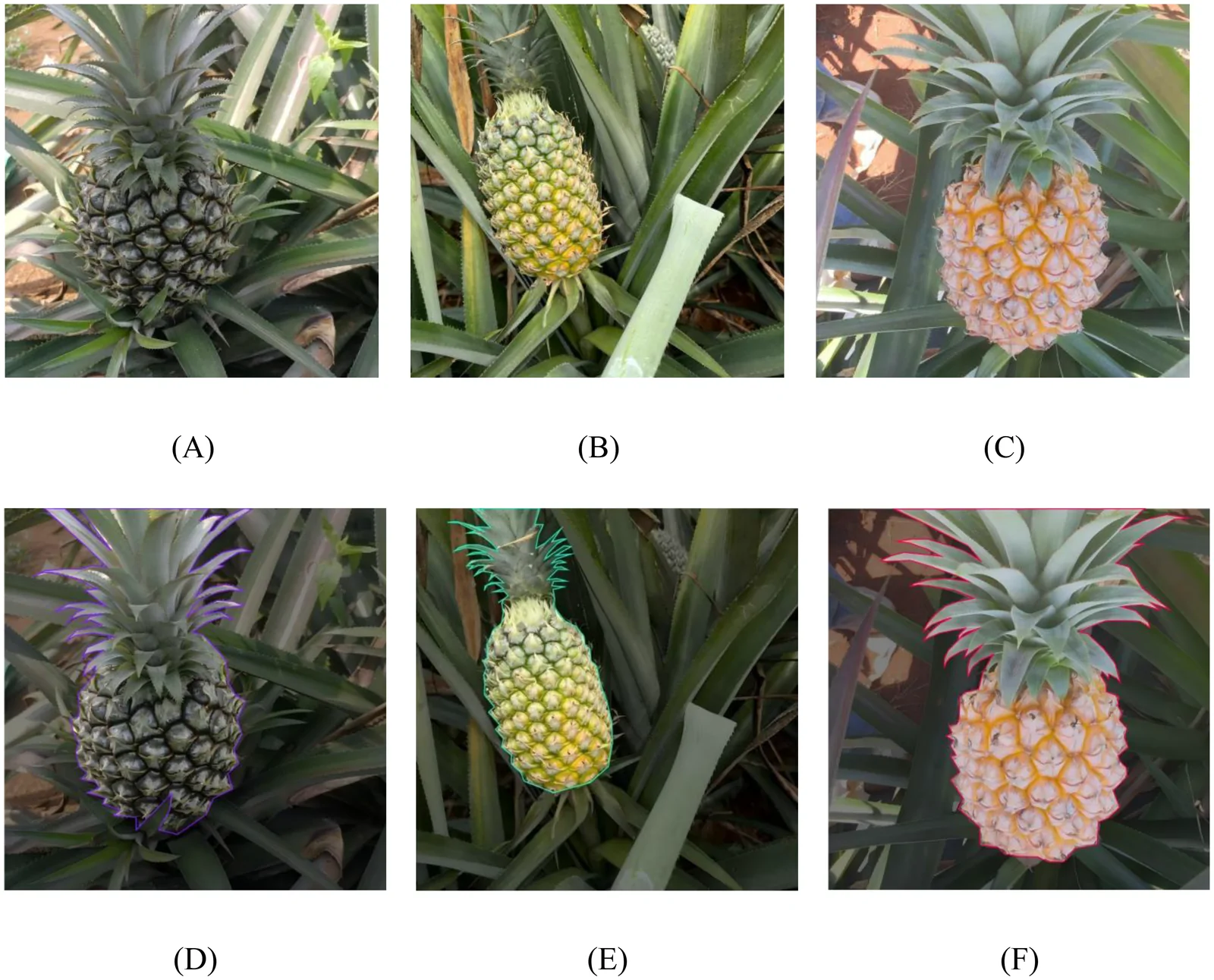
Representative pineapples at different appearance-based ripeness stages and their corresponding polygon annotations. **(A)** Unripe pineapple. **(B)** Partially ripe pineapple. **(C)** Ripe pineapple. **(D–F)** Corresponding ground-truth polygon annotations.

**Table 2:** Visual grading and quantitative criteria for appearance-based pineapple ripeness.

Grade	Ripeness label	Principal phenotype and visual characteristics	Yellowed-area proportion (r)	Corresponding NY/T 450–2001 stage
1	Unripe	Pineapple eyes are well developed; the fruit surface is predominantly green, and the grooves may show slight yellowing.	r ≤ 5%	Stage 1
2	Partially ripe	Color change begins at the fruit base; green and yellow regions coexist, and the yellowed region gradually extends upward.	5%<r<50%	Stages 2 and 3
3	Ripe	At least approximately half of the visible fruit surface is yellow or orange-yellow, and yellow coloration gradually becomes dominant.	r≥50%	Stage 4

To improve the consistency of ripeness labeling, the yellowed-area proportion of the visible fruit surface was used as an auxiliary criterion and was calculated as shown in Equation 1:

(1) $$r=\frac{A_{\text{y}}}{A_{\text{f}}}\times 100$$

where *A_y_* is the pixel area of the yellow or orange-yellow region on the visible pineapple surface, *A_f_* is the total pixel area of the visible pineapple surface, and *r* is the yellowed-area proportion of the visible surface. Based on the surface-color descriptions in the standard and the practical requirements of annotation, (r ≤ 5%), (5%< r< 50%), and (r ≥ 50%) were used as auxiliary ranges for the Unripe, Partially ripe, and Ripe classes, respectively.

NY/T 450–2001 also states that surface color does not always fully reflect internal physiological maturity and that the translucency of the flesh in cross-section provides a more reliable assessment. Given the constraints of non-contact RGB imaging in the field, the detection target in this study was defined as appearance-based ripeness rather than internal physiological maturity based on flesh condition. The yellowed-area proportion served only as a visual reference during dataset annotation and was not used in PineRipeSeg inference. The model directly output an instance mask, bounding box, confidence score, and ripeness class for each pineapple.

### 2.4 Dataset construction

The screened pineapple images were initially processed using the automatic annotation function of the Roboflow platform to generate preliminary polygon masks. One annotator then reviewed and corrected each preliminary mask image by image and verified the corresponding appearance-based ripeness label against the original image and the criteria described in Section 2.3 and **Table 2**. For samples whose ripeness class could not be confidently determined by direct visual assessment, the visible pineapple surface was manually selected, and an auxiliary program was used to calculate the proportion of yellow or orange-yellow pixels within the selected region. The final ripeness class was then assigned according to the thresholds specified in **Table 2**. This procedure was applied consistently to visually ambiguous samples near the class boundaries to reduce subjective variation in the final labels. However, because all final masks and ripeness labels were reviewed by a single annotator, inter-annotator agreement was not quantified. This remains a limitation of the present dataset. The annotations were saved as TXT files in the YOLO instance-segmentation format. Each label contained a class index and normalized polygon-contour coordinates. The classes were Unripe, Partially ripe, and Ripe.

An occlusion-aware hybrid annotation protocol was adopted for pineapple masks. When a fruit was occluded only by narrow leaves and the hidden contour could be reliably inferred from the visible boundaries on both sides and the continuous morphology of the fruit, the mask was extended across the occluded region to approximate the complete fruit extent. When the hidden contour could not be inferred with sufficient confidence because of severe or extensive occlusion, only the actually visible fruit region was annotated. Therefore, the dataset contains visible-region masks for uncertain occlusions and partially amodal masks for locally recoverable occlusions. Pixels corresponding to foreground leaves within a partially amodal mask represent the inferred spatial extent of the occluded fruit rather than its directly visible surface. This protocol was intended to preserve the fruit center, overall extent, and contour continuity for subsequent target localization while avoiding arbitrary completion of highly uncertain regions.

Consecutive acquisition of field images can produce strong feature overlap among samples from the same source. Conventional random proportional splitting can therefore cause data leakage and inflate test accuracy (Roberts et al., 2017). To address this risk, the dataset was independently divided using a cross-domain grouping strategy. Images acquired in the same plot and year were not allowed to appear simultaneously in the training, validation, and test sets. This physical separation by acquisition year and spatial plot was intended to provide a more objective assessment of generalization under an unseen domain shift.

The resulting field pineapple instance-segmentation dataset contained 6,019 valid images: 4,213 training images, 903 validation images, and 903 test images. At the polygon-instance level, the dataset contained 9,393 pineapple annotations, comprising 2,909 Unripe, 2,892 Partially ripe, and 3,592 Ripe instances. The class distribution was generally balanced and provided a reliable basis for training and evaluating PineRipeSeg.

### 2.5 Development of PineRipeSeg

#### 2.5.1 Overall network architecture

YOLO11 is a recent model in the YOLO series released by Ultralytics and includes optimizations for feature extraction, multiscale feature fusion, and computational efficiency (Luo et al., 2025; Shi et al., 2025). Its backbone uses C3k2 modules to extract image features, while SPPF and C2PSA modules expand the receptive field and enhance high-level semantic information. The neck fuses features at different scales to support multiscale target recognition in complex backgrounds. In addition to the object-detection branch, YOLO11-seg contains an instance-segmentation branch and can simultaneously output the target class, bounding box, confidence score, and instance mask (Luo et al., 2025). YOLO11n-seg was selected as the baseline because it combines relatively low model complexity with high inference efficiency.

PineRipeSeg was then developed to address three limitations of field pineapple instance segmentation. TEDA was designed to reduce fruit–leaf feature confusion by preserving pineapple-eye texture and attenuating elongated linear structures. GhostConv was introduced into the backbone to reduce redundant computation caused by repeated fruit-eye patterns and leaf-edge features. Inner-IoU was applied to the bounding-box regression branch to improve the localization of partially visible fruit under severe occlusion. Thus, the three components perform distinct functions in feature discrimination, computational efficiency, and localization refinement rather than forming a simple stack of optimization modules. The overall architecture of PineRipeSeg is shown in **Figure 5**.

**Figure 5:**
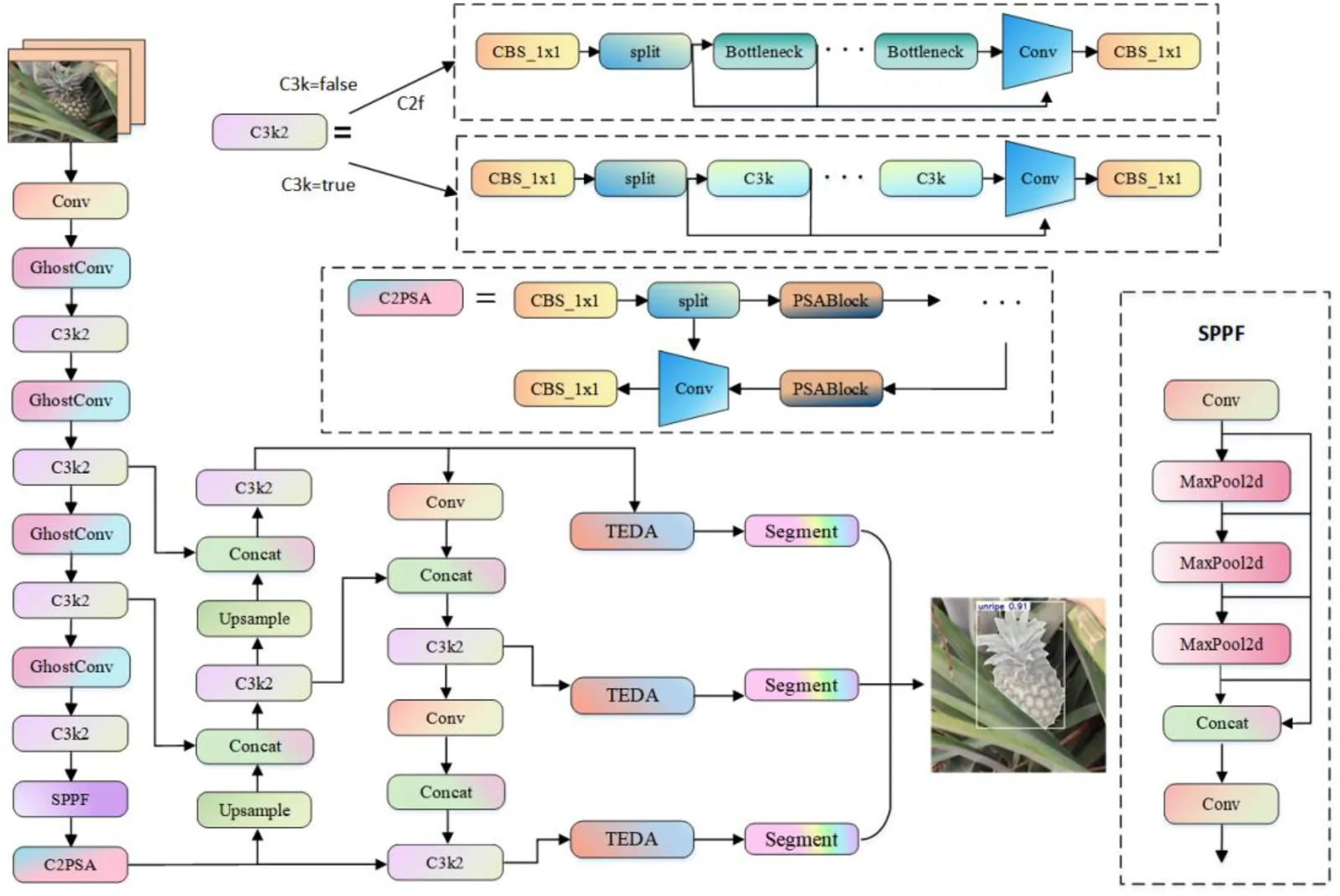
Network architecture of PineRipeSeg.

#### 2.5.2 Texture-edge decoupling attention

Field pineapple images contain a task-specific structural conflict between fruit and surrounding foliage. Pineapple eyes form repeated and intersecting boundaries distributed across multiple orientations, whereas sword-shaped leaves and crowns are dominated by elongated edges with a more pronounced principal direction. This difference becomes particularly important when color information is ambiguous because green leaves can resemble Unripe fruit and senescent yellow leaves can resemble Ripe fruit. General-purpose spatial or positional attention mechanisms can recalibrate salient feature responses, but they do not explicitly model this crop-specific texture–edge contrast. TEDA was therefore designed on the basis of this structural difference to strengthen the representation of multidirectional pineapple-eye texture while reducing interference from predominantly linear leaf structures.

As shown in **Figure 6**, TEDA was independently inserted into the P3, P4, and P5 feature branches before the features entered the unified segmentation head. The three modules use the same computational structure but have independent parameters. For the feature branch *P_t_*, where t∈{3,4,5}, let *X_t_* denote the input feature map with dimensions *C_t_* × *H_t_* × *W_t_*, where *C_t_*, *H_t_*, and *W_t_* denote the channel number, height, and width, respectively. In Equations 2, 3, *c* denotes the channel index, while *i* and *j* denote the spatial position. Channel-wise average pooling and maximum pooling are first performed:

**Figure 6:**
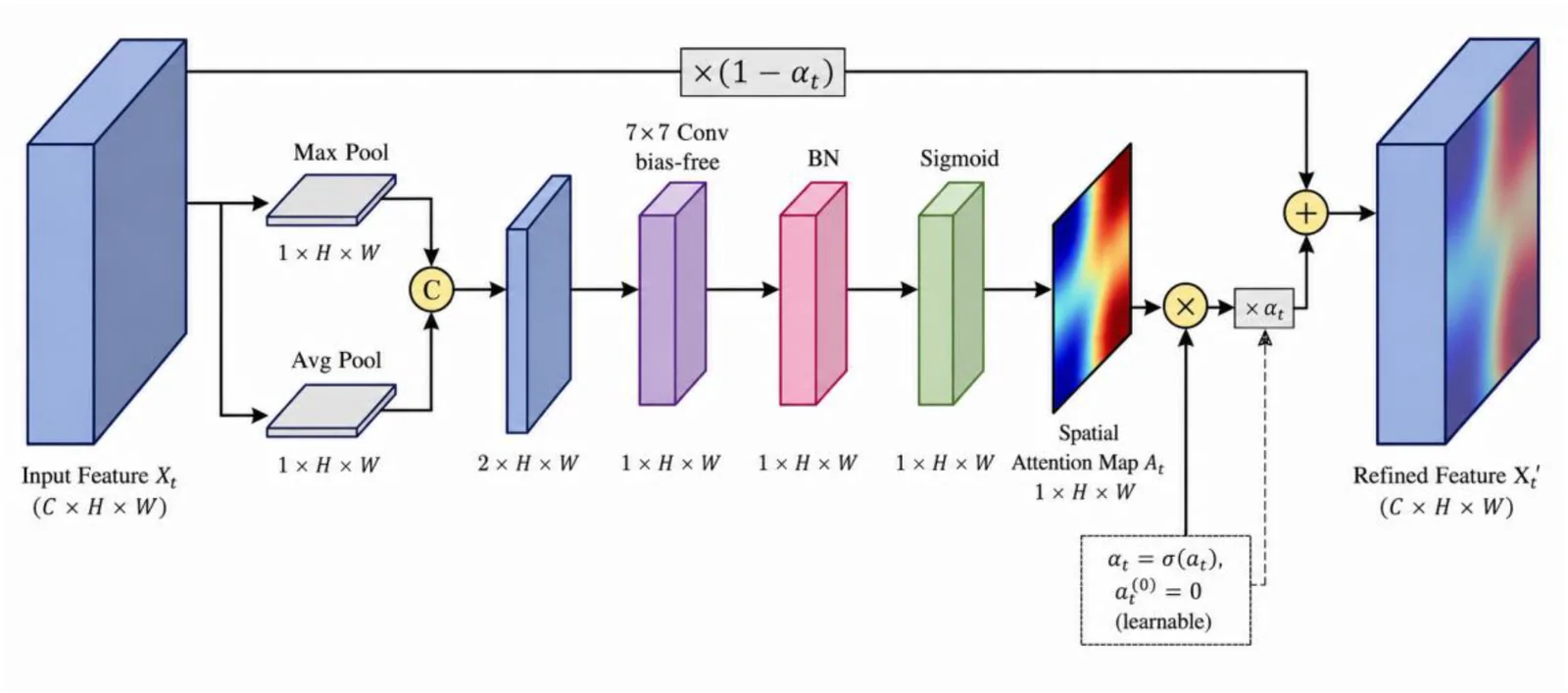
Architecture of the TEDA module.

(2) $$F_{\text{avg},t}\left(i,j\right)=\frac{1}{C_{t}}\sum_{c=1}^{C_{t}}X_{t}\left(c,i,j\right)$$

(3) $$F_{\max,t}\left(i,j\right)=\underset{1\leq c\leq C_{t}}{\max}X_{t}\left(c,i,j\right)$$

Average pooling describes the overall response across feature channels, whereas maximum pooling retains the strongest response at each spatial position. Both operations aggregate responses across channels while preserving spatial information. The two pooled descriptors have dimensions 1×Ht×Wt and provide complementary spatial information for the subsequent attention calculation and structural analysis.

The structural difference between pineapple eyes and leaf edges can be characterized by their local gradient distributions. Pineapple-eye regions contain intersecting boundaries with multiple orientations, whereas elongated leaf edges are usually dominated by one principal direction. To describe this distinction theoretically, the local structure tensor is used as an analytical representation of the directional distribution within the pooled feature descriptors (Harris and Stephens, 1988). In Equation 4, *p* denotes the center position of the local neighborhood, *q* denotes a position within that neighborhood, and the subscript *u* refers to either the average-pooled or maximum-pooled descriptor. The local structure tensor is a 2×2 matrix constructed from the horizontal and vertical gradients within this neighborhood:

(4) $$J_{t}\left(p\right)=\sum_{q\in \Omega_{7\times 7}\left(p\right)}g\left(q-p\right)\sum_{u\in \left\{avg,\max\right\}}\nabla F_{u,t}\left(q\right)\nabla F_{u,t}{\left(q\right)}^{T}$$

where ${\text{Ω}}_{7\times 7}\left(p\right)$ denotes the local neighborhood centered at *p*, and *g*(*q* − *p*) is a local spatial weighting function. The vector $\nabla F_{u,t}\left(q\right)$ contains the horizontal and vertical gradients of the pooled descriptor. Let $\lambda_{1,t}\left(p\right)$ and $\lambda_{2,t}\left(p\right)$ be the two eigenvalues of *J_t_*(*p*), with $\lambda_{1,t}\left(p\right)\geq \lambda_{2,t}\left(p\right)\geq 0$. The directional anisotropy of the local structure is expressed as:

(5) $$D_{t}\left(p\right)=\frac{\lambda_{1,t}\left(p\right)-\lambda_{2,t}\left(p\right)}{\lambda_{1,t}\left(p\right)+\lambda_{2,t}\left(p\right)+\epsilon }$$

where *ϵ* is a small constant used to prevent division by zero. A long leaf edge is usually dominated by one gradient direction. It therefore tends to produce one large eigenvalue and one relatively small eigenvalue, resulting in a high *D_t_*. Pineapple-eye regions contain several intersecting or interlocking boundaries. Their gradient energy is distributed over multiple directions, and both eigenvalues are generally non-negligible. Consequently, these regions usually exhibit lower directional anisotropy than isolated leaf edges.

Equations 4, 5 provide an analytical description of the local directional difference that motivates TEDA; they are not part of the actual forward computation of the module. In particular, TEDA does not explicitly calculate the structure tensor, its eigenvalues, or the directional-anisotropy measure during inference. Instead, the implemented module learns spatial responses directly from the pooled feature descriptors through the trainable convolution described in Equations 6–11. The convolution-based implementation is therefore inspired by this structural distinction rather than being a mathematical approximation of the structure tensor.

The average- and maximum-pooled descriptors are concatenated along the channel dimension. A bias-free 7×7 convolution is then applied. The 7×7 neighborhood provides a relatively broad spatial context. It can cover multiple adjacent pineapple-eye boundaries and their directional changes. It can also capture the longer and more continuous spatial pattern of leaf edges (Ding et al., 2022). The convolutional weights are jointly optimized by the task losses. They gradually learn different responses to these structural patterns.

The convolution output is processed by Batch Normalization and a sigmoid activation function to generate the spatial attention map:

(6) $$A_{t}=\sigma \left(BN_{t}\left[f_{\text{conv},t}^{7\times 7}\left(Cat\left(F_{\text{avg},t},F_{\max,t}\right)\right)\right]\right)$$

where Cat(·) denotes channel concatenation. The convolution has two input channels and one output channel. Its padding is 3, and no bias is used. Batch Normalization contains learnable scale and shift parameters (Ioffe and Szegedy, 2015). It stabilizes the distribution of the convolution output during training and reduces the risk of premature saturation of the sigmoid function. The resulting attention map has dimensions 1 × *H_t_* × *W_t_* and is broadcast across the channel dimension when applied to the input feature map. The refined feature produced by Equation 8 retains the same dimensions *C_t_* × *H_t_* × *W_t_* as the input feature map, while the spatial gain in Equation 9 has dimensions 1 × *H_t_* × *W_t_*.

A learnable scalar *a_t_* is assigned independently to each feature branch. The effective fusion coefficient is calculated as:

(7) $$\alpha_{t}=\sigma \left(a_{t}\right),a_{t}^{\left(0\right)}=0,\alpha_{t}^{\left(0\right)}=0.5$$

The raw parameter *a_t_* is initialized to zero. The initial fusion coefficient is therefore 0.5. This initialization assigns equal initial scalar mixing coefficients to the original feature branch and the attention-filtered branch. During training, the three fusion coefficients are optimized independently according to the supervision received at each feature scale.

The refined feature is calculated as:

(8) $$X_{t}^{\prime }=\left(1-\alpha_{t}\right)X_{t}+\alpha_{t}\left(X_{t}⊗A_{t}\right)$$

where ⊗ denotes element-wise multiplication with channel-wise broadcasting. The fusion process can also be expressed using an equivalent spatial gain:

(9) $$G_{t}=1-\alpha_{t}\left(1-A_{t}\right)$$

Accordingly,

(10) $$X_{t}^{\prime }=X_{t}⊗G_{t}$$

Since 0< *A_t_* < 1 and 0< *α_t_* < 1, the spatial gain satisfies:

(11) $$1-\alpha_{t}<G_{t}<1$$

The inequalities in Equation 11 apply element-wise. Regions with high attention values retain a larger proportion of the original response. Regions dominated by leaf-related linear structures receive stronger attenuation. TEDA therefore improves the relative prominence of pineapple-eye texture while retaining a direct propagation path for the original features. This design reduces the risk that weak but valid fruit regions will be excessively suppressed under severe occlusion.

The three TEDA instances learn independent 7×7 convolutional weights, Batch Normalization parameters, and fusion coefficients. This non-shared design accommodates the different spatial resolutions of P3, P4, and P5. The pixel span of pineapple-eye texture changes across these feature levels. In addition, the same 7×7 kernel corresponds to a different effective image region at each scale. Independent parameter learning therefore enables scale-specific texture-edge response calibration before the multiscale features enter the segmentation head.

Each TEDA instance contains 98 convolutional weights, two Batch Normalization affine parameters, and one residual-scaling parameter. The three instances introduce only 303 learnable parameters in total. Their parameter count is independent of the number of input channels because channel pooling reduces the input to two spatial descriptors. Through this lightweight design, TEDA improves the relative representation of multidirectional fruit texture and suppresses predominantly linear leaf responses without substantially increasing network complexity.

#### 2.5.3 GhostConv lightweight feature-extraction module

For automated field harvesting, visual-perception models may ultimately need to operate on agricultural platforms with limited computing capacity and power budgets. Lightweight backbones and efficient convolutional structures have therefore been explored to improve the real-time performance of agricultural vision models (Ji W. et al., 2022; Wang A. et al., 2025). However, repeated pineapple-eye patterns and elongated leaf edges can still produce correlated feature maps during low- and middle-level feature extraction, increasing redundant computation.

The lightweight GhostConv module (Han et al., 2020; Wang X. and Liu, 2024) was introduced to reconstruct the network’s feature-extraction pathway. GhostConv exploits redundancy in deep feature maps by replacing part of standard convolution with low-cost linear transformations. This design reduces computation while retaining high-dimensional semantic information. Its operation comprises two stages (**Figure 7**). First, a small number of standard convolution kernels generate intrinsic feature maps. Low-cost linear operations, such as 3 × 3 or 5 × 5 depthwise convolutions, then expand these maps channel by channel. Finally, the intrinsic and expanded feature maps are concatenated to produce an output with the same dimensions as standard convolution. The GhostConv formulation uses *h*, *w*, and *c* to denote the input height, width, and number of channels, respectively. *H* and *W* denote the output height and width. The total number of output feature maps and the number of intrinsic feature maps are denoted by *n* and *m*, respectively. Each intrinsic feature map generates additional feature maps through the low-cost transformations, and the relationship used in Equations 12, 13 is *n* = *ms*. The kernel sizes of the standard convolution and linear transformation are denoted by *k* and *d*, while s denotes the number of transformations. The theoretical ratios of computational complexity and parameter count between standard convolution and GhostConv are given in Equations 12, 13:

**Figure 7:**
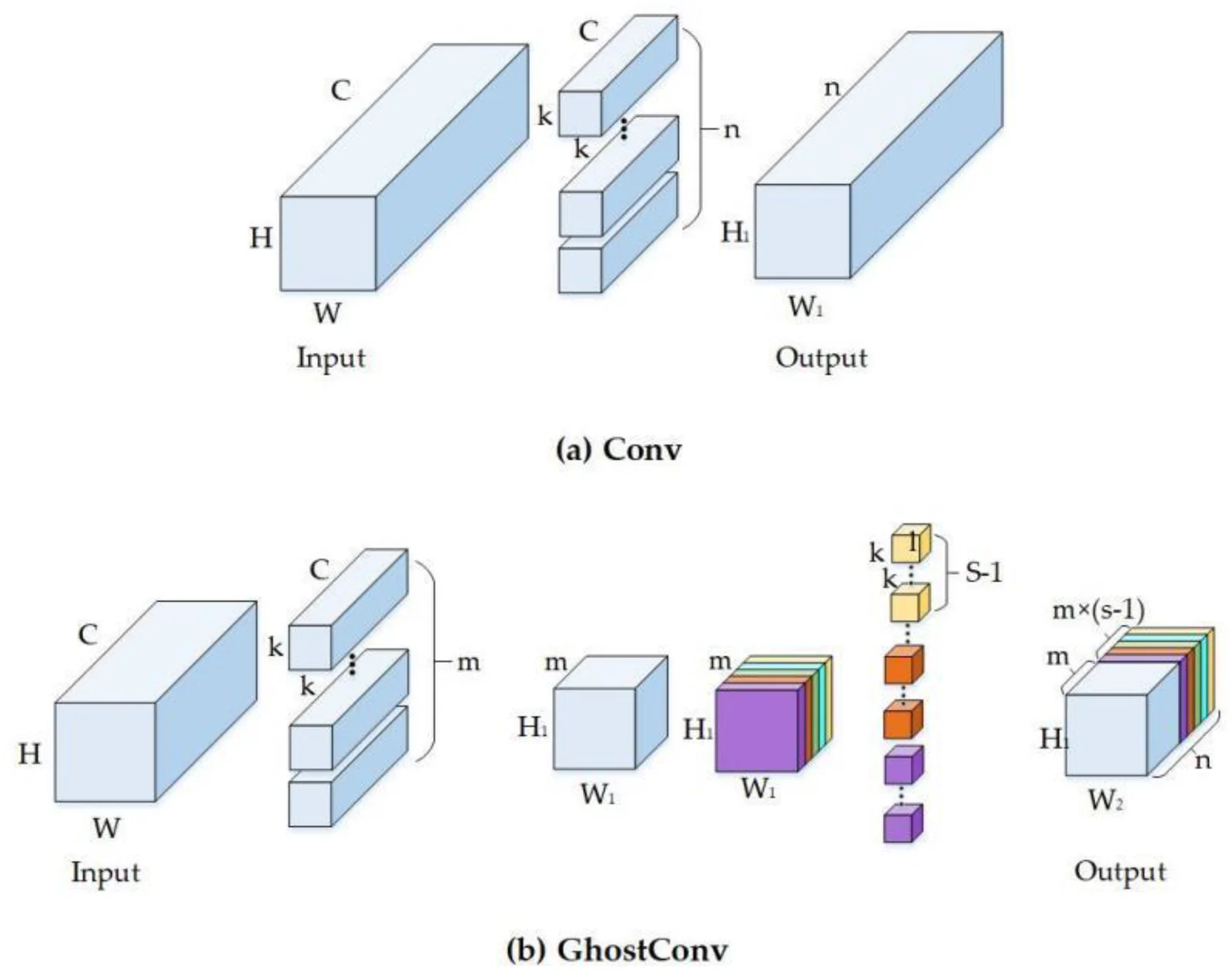
Structure of the GhostConv module. **(a)** Standard convolution (Conv). **(b)** Ghost convolution (GhostConv).

(12) $$r_{s}=\frac{n\cdot H\cdot W\cdot c\cdot k^{2}}{m\cdot H\cdot W\cdot c\cdot k^{2}+m\cdot \left(s-1\right)\cdot H\cdot W\cdot d^{2}}\approx \frac{s\cdot c}{c+s-1}\approx s$$

(13) $$r_{c}=\frac{n\cdot c\cdot k^{2}}{m\cdot c\cdot k^{2}+m\cdot \left(s-1\right)\cdot d^{2}}\approx \frac{s\cdot c}{c+s-1}\approx s$$

Equations 12, 13 show that the ratios of computation and parameter count are closely related to the number of transformations, denoted by s. The acceleration becomes more pronounced as the number of generated feature maps increases. Introducing GhostConv can therefore reduce both computation and the number of parameters.

In PineRipeSeg, GhostConv replaced the standard convolutional modules in the backbone, except for the initial convolution. Field pineapple images contain repeated pineapple-eye patterns and numerous elongated leaf edges, which can produce highly correlated feature maps during low- and middle-level feature extraction. GhostConv reduces this redundancy by generating part of the feature representation through low-cost linear operations. The original efficient convolutional structures were retained in the neck because this stage performs multiscale semantic fusion and is more sensitive to reductions in feature capacity. GhostConv was therefore used specifically to reduce backbone computation while preserving the feature-fusion ability required for pineapple instance segmentation and ripeness classification.

#### 2.5.4 Inner-IoU bounding-box regression loss

Under field imaging conditions, pineapple fruits are frequently occluded by leaves. The resulting incomplete visible contours can cause errors in the position and scale of predicted boxes. YOLO11n-seg uses Complete Intersection over Union (CIoU) for bounding-box regression. CIoU considers the overlap area, center-point distance, and aspect ratio between the predicted and ground-truth boxes. However, its intersection-over-union (IoU) term is always calculated from the original boxes and cannot adjust the effective gradient range across regression stages. Inner-IoU was therefore introduced into the bounding-box regression branch to improve pineapple localization. Published agricultural object-detection research has demonstrated that combining Inner-IoU with CIoU enables the overlap term to be calculated using scaled auxiliary boxes and can improve bounding-box regression accuracy (Li Y. et al., 2024).

Let the predicted and ground-truth boxes be $\left(B_{p}=\left(x_{p},y_{p},w_{p},h_{p}\right)\right)$ and $\left(B_{g}=\left(x_{g},y_{g},w_{g},h_{g}\right)\right)$, respectively, where (*x*) and (*y*) denote the center coordinates and (*w*) and (*h*) denote the width and height, respectively. With the box centers fixed, the corresponding inner auxiliary boxes are constructed using the scale factor *η*, as shown in Equation 14:

(14) $$B_{p}^{\text{in}}=\left(x_{p},y_{p},\eta w_{p},\eta h_{p}\right),\;B_{g}^{\text{in}}=\left(x_{g},y_{g},\eta w_{g},\eta h_{g}\right)$$

where (*η*) is the auxiliary-box scale factor. The intersection over union of the auxiliary predicted and ground-truth boxes is calculated as follows:

(15) $${\text{IoU}}_{\text{inner}}=\frac{\left|B_{p}^{\text{in}}\cap B_{g}^{\text{in}}\right|}{\left|B_{p}^{\text{in}}\cup B_{g}^{\text{in}}\right|}$$

When (*η* < 1), the auxiliary boxes are smaller than the original boxes. For high-IoU samples, smaller auxiliary boxes produce larger IoU gradients and can strengthen fine-stage bounding-box regression (Cui et al., 2026). In agricultural vision research, Chen et al. (2026) also selected a scale factor of 0.8 for Inner-CIoU after systematic experimentation in orchard pear detection. Based on this regression mechanism and the reported empirical settings, *η* was fixed at 0.8 in the present study.

Equation 15 calculates the overlap of the scaled auxiliary predicted and ground-truth boxes, whereas the standard IoU term is calculated from the original predicted and ground-truth boxes. Inner-IoU replaces this original overlap term while retaining the center-distance and aspect-ratio constraints of CIoU, yielding the Inner-CIoU loss given in Equation 16:

(16) $$L_{\text{Inner-CIoU}}=L_{\text{CIoU}}+\text{IoU}-{\text{IoU}}_{\text{inner}}$$

Inner-IoU acts only on the bounding-box regression branch of PineRipeSeg. Severe leaf occlusion leaves incomplete fruit regions and makes box regression more sensitive to small errors in position and scale. With *η* set to 0.8, the auxiliary boxes remain smaller than the original boxes, emphasizing the refinement of high-IoU samples. This helps the predicted boxes approach the annotated extent of the fruit more closely. More accurate box localization also provides a more reliable spatial extent for subsequent instance-mask processing. The classification and mask-prediction structures remain unchanged. Inner-IoU therefore complements TEDA: TEDA improves fruit–leaf feature discrimination, whereas Inner-IoU refines target localization.

### 2.6 Experimental environment and evaluation metrics

To maintain a controlled comparison, all models were trained and tested using the same dataset split, principal training settings, hardware and software environment, and evaluation protocol. The configuration is provided in **Table 3**.

**Table 3:** Software and hardware environment and principal training parameters.

Category	Configuration item	Specification
Hardware	CPU	15 vCPU Intel(R) Xeon(R) Platinum 8474C
	Memory	120 GB
	GPU	NVIDIA GeForce RTX 4090D, 24 GB
Software	Operating system	Ubuntu 18.04
	CUDA version	11.8
	Python version	3.8.1
	Deep-learning framework	PyTorch 2.0.0
Training parameters	Epochs	300
	Batch size	32

To evaluate PineRipeSeg comprehensively and objectively under complex field conditions, a structured set of metrics was established across three dimensions: target localization and mask segmentation, ripeness-class classification, and computational efficiency.

(1) Target-localization and mask-segmentation metrics. Mean average precision (mAP) was used as the primary metric for spatial localization and boundary prediction in the instance-segmentation task. Bounding-box metrics (Box mAP) and mask metrics (Mask mAP) were calculated separately to quantify overall fruit localization and detailed contour prediction. mAP50 was calculated at an intersection-over-union (IoU) threshold of 0.5, whereas mAP50–95 was averaged over IoU thresholds from 0.5 to 0.95 in increments of 0.05. The latter metric emphasizes the detailed boundary fit of occluded targets.

(2) Ripeness-class classification metrics. Precision (P), recall (R), and the F1-score (*F*_1_) were used to assess discrimination among the Unripe, Partially ripe, and Ripe appearance classes. These metrics were calculated using Equations 17–19:

(17) $$P=\frac{\text{TP}}{\text{TP}+\text{FP}}$$

(18) $$R=\frac{\text{TP}}{\text{TP}+\text{FN}}$$

(19) $$F_{1}=\frac{2PR}{P+R}$$

where *TP* (true positives) is the number of pineapple instances correctly identified as positive samples, *FP* (false positives) is the number of negative samples incorrectly identified as positive samples, and *FN* (false negatives) is the number of positive samples missed by the model.

(3) Computational-efficiency metrics. Three metrics were used to characterize model complexity and inference efficiency: GFLOPs, model file size, and FPS on the specified experimental platform. GFLOPs quantify the theoretical computational cost of one forward pass and the associated demand for computing resources. Model file size, reported in megabytes (MB), reflects the storage footprint of the trained model. FPS measures the inference throughput on the specified hardware platform.

For the repeated comparison of attention mechanisms, differences between TEDA and CBAM in Box mAP50 and Mask mAP50 were evaluated using two-tailed paired t-tests across the five training runs matched by random seed. For each paired test, the degrees of freedom (df) were 4. Statistical significance was defined at p< 0.05.

## 3 Results

### 3.1 Model-training results

To examine the convergence of PineRipeSeg, the performance metrics and loss functions were recorded over 300 training epochs (**Figure 8**). During the early training stage, precision, recall, mAP50, and mAP50–95 for bounding-box detection and instance-mask segmentation increased rapidly with some fluctuation. As training progressed, variation in these metrics decreased and the curves stabilized. Bounding-box regression loss, instance-segmentation loss, classification loss, and distribution focal loss decreased rapidly at first and then more gradually. During the final training stage, the loss curves remained stable without pronounced oscillation or abnormal increases. These trends indicated stable training and convergence.

**Figure 8:**
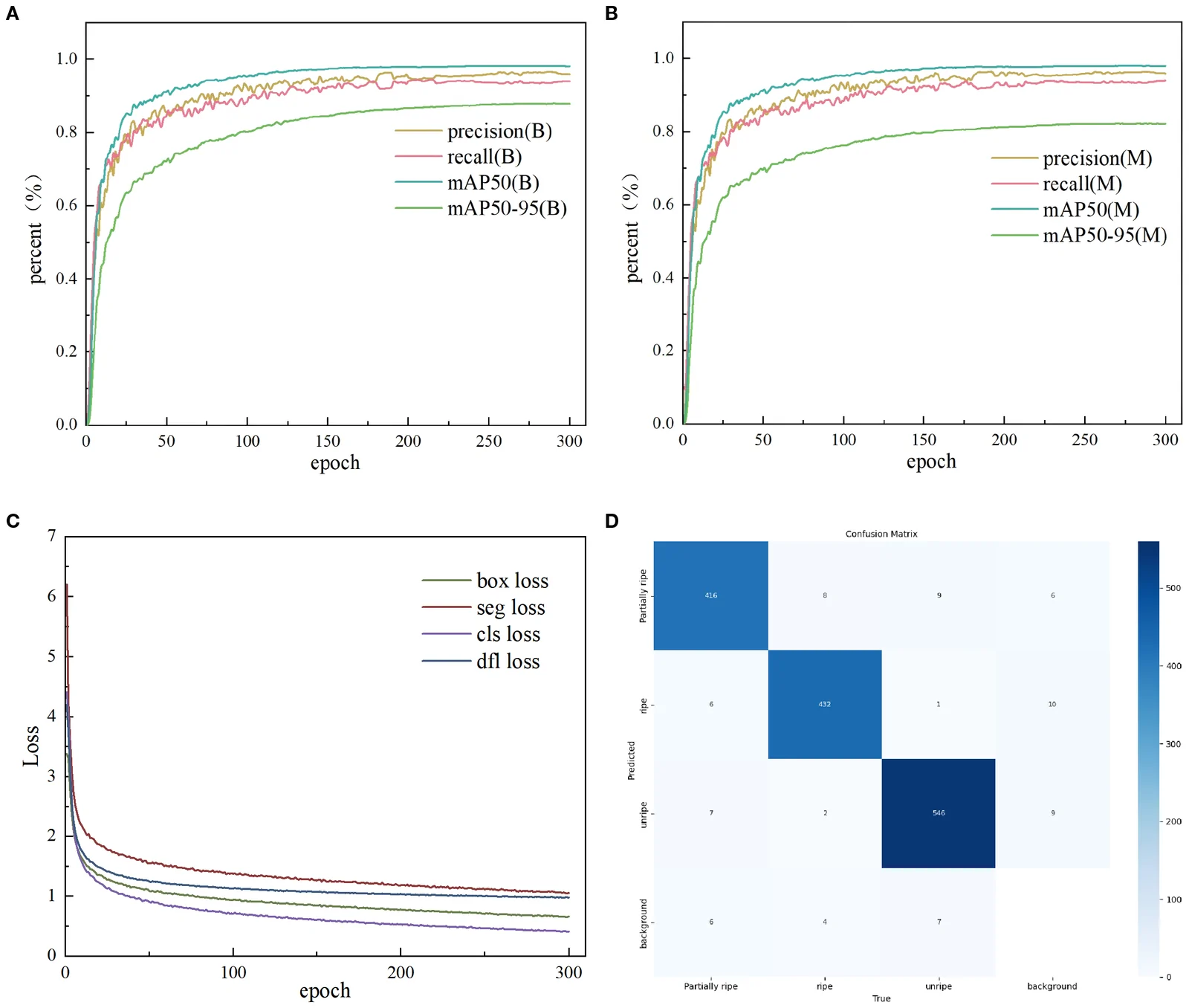
Training results for PineRipeSeg. **(A)** Bounding-box precision, recall, mAP50, and mAP50–95. **(B)** Instance-mask precision, recall, mAP50, and mAP50–95. **(C)** Bounding-box regression loss, segmentation loss, classification loss, and distribution focal loss. **(D)** Confusion matrix on the validation set.

Using the best weights saved during training, PineRipeSeg achieved bounding-box precision, recall, F1-score, mAP50, and mAP50–95 values of 0.964, 0.937, 0.950, 0.981, and 0.880, respectively. The corresponding instance-mask segmentation values were 0.964, 0.937, 0.950, 0.980, and 0.822. The computational cost was 8.7 GFLOPs, the model file size was 5.1 MB, and the inference speed on the experimental platform was 121 FPS. These results show that PineRipeSeg achieved high accuracy in pineapple localization, occlusion-aware hybrid instance segmentation, and appearance-based ripeness classification while controlling computation and model size. The confusion matrix in **Figure 8D** further shows that most samples were correctly classified, with misclassifications occurring mainly between Partially ripe and the adjacent ripeness classes.

### 3.2 Performance comparison with other models

To evaluate the competitiveness of PineRipeSeg across representative instance-segmentation architectures, it was compared with Mask R-CNN (He K. et al., 2017), Mask2Former (Cheng et al., 2022), YOLOv5s-seg, YOLOv8n-seg, and YOLO11n-seg. These models cover different architectural paradigms: Mask R-CNN represents a classical two-stage convolutional instance-segmentation framework, Mask2Former represents a Transformer-based segmentation architecture, and YOLOv5s-seg, YOLOv8n-seg, and YOLO11n-seg represent different generations of one-stage real-time instance-segmentation models. All models were trained and evaluated using the same dataset split, principal training settings, evaluation protocol, and experimental environment to maintain a controlled comparison. The results are presented in **Table 4**. Each model in **Table 4** was evaluated based on a single training run.

**Table 4:** Performance comparison among instance-segmentation models.

Model	Box mAP50	Mask mAP50	Mask mAP50–95	GFLOPs	FPS
Mask R-CNN	–	0.915	0.773	121	16
Mask2Former	–	0.932	0.792	107	19
YOLOv5s-seg	0.916	0.870	0.716	18.2	95
YOLOv8n-seg	0.927	0.892	0.751	10.1	107
YOLO11n-seg	0.931	0.928	0.788	9.6	110
PineRipeSeg	0.981	0.980	0.822	8.7	121

As shown in **Table 4**, PineRipeSeg achieved the highest mask-segmentation accuracy among the evaluated models. Mask R-CNN obtained Mask mAP50 and Mask mAP50–95 values of 0.915 and 0.773, respectively, while Mask2Former achieved corresponding values of 0.932 and 0.792. PineRipeSeg increased these values to 0.980 and 0.822. Among the YOLO-based models, PineRipeSeg also achieved the highest Box mAP50, Mask mAP50, and Mask mAP50–95. Compared with the YOLO11n-seg baseline, the corresponding values increased by 5.0, 5.2, and 3.4 percentage points, respectively. These results indicate improved pineapple localization and more accurate segmentation of the annotated pineapple instance contours.

Mask R-CNN and Mask2Former provided competitive segmentation accuracy, but their computational costs reached 121 and 107 GFLOPs, respectively, and their inference speeds were only 16 and 19 FPS. Their relatively high computational requirements and low inference throughput resulted in a less favorable balance between segmentation accuracy and computational efficiency. In contrast, PineRipeSeg required only 8.7 GFLOPs and achieved an inference speed of 121 FPS. Compared with YOLO11n-seg, its computational cost decreased from 9.6 to 8.7 GFLOPs, while the inference speed increased from 110 to 121 FPS. These findings demonstrate that PineRipeSeg achieved a more favorable balance between segmentation accuracy, computational cost, and inference efficiency than the evaluated convolutional, Transformer-based, and YOLO-based instance-segmentation models.

### 3.3 Ablation experiments

To evaluate the effects of TEDA, GhostConv, and Inner-IoU, ablation experiments were conducted using YOLO11n-seg as the baseline, while the dataset, training parameters, and experimental environment were held constant. Each of the three modifications was first introduced separately. Pairwise and complete combinations were then evaluated, producing eight experimental configurations, as shown in **Table 5**. Each configuration in **Table 5** was evaluated based on a single training run.

**Table 5:** Ablation results for PineRipeSeg.

Model	Box P	Box R	Box mAP50	Box mAP50–95	Mask P	Mask R	Mask mAP50	Mask mAP50–95	GFLOPs	Model file size	FPS
YOLO11n-seg	0.906	0.897	0.931	0.843	0.905	0.897	0.928	0.788	9.6	6.0 MB	110
YOLO11n-seg-T	0.946	0.925	0.966	0.861	0.947	0.925	0.966	0.810	9.6	6.0 MB	103
YOLO11n-seg-G	0.909	0.885	0.932	0.829	0.909	0.885	0.932	0.771	8.7	5.1 MB	125
YOLO11n-seg-I	0.934	0.917	0.956	0.873	0.931	0.916	0.950	0.808	9.6	6.0 MB	110
YOLO11n-seg-TG	0.942	0.922	0.963	0.857	0.943	0.922	0.962	0.806	8.7	5.1 MB	121
YOLO11n-seg-TI	0.960	0.935	0.977	0.882	0.961	0.934	0.976	0.820	9.6	6.0 MB	103
YOLO11n-seg-GI	0.930	0.914	0.953	0.869	0.928	0.913	0.947	0.804	8.7	5.1 MB	125
PineRipeSeg	0.964	0.937	0.981	0.880	0.964	0.937	0.980	0.822	8.7	5.1 MB	121

T denotes TEDA, G denotes GhostConv, and I denotes Inner-IoU.

As shown in **Table 5**, TEDA produced a clear improvement in pineapple instance segmentation. After TEDA was introduced, Box mAP50 and Mask mAP50 increased by 3.5 and 3.8 percentage points, respectively, while Mask mAP50–95 increased by 2.2 percentage points. These results indicate that TEDA exploited the structural difference between the multidirectional interlocking texture of pineapple eyes and the predominantly linear edges of leaves. It reduced interference from sword-shaped leaf occlusion and similarly colored backgrounds and enhanced texture representation and boundary discrimination on the visible fruit surface. Inner-IoU mainly improved bounding-box regression: Box mAP50–95 increased by 3.0 percentage points, accompanied by an increase in Mask mAP50–95. More accurate localization therefore provided more reliable spatial constraints for instance-mask generation. GhostConv substantially reduced computational cost and model file size while increasing inference speed, indicating that it reduced the computational burden while retaining the principal feature-representation capacity.

The combined configurations further revealed distinct interactions among the three modifications. Among the individual modules, TEDA produced the largest improvements in Box mAP50 and Mask mAP50, whereas Inner-IoU produced the largest improvement in Box mAP50–95; GhostConv mainly reduced computational cost and model size. Combining TEDA and Inner-IoU increased Box mAP50, Box mAP50–95, Mask mAP50, and Mask mAP50–95 to 0.977, 0.882, 0.976, and 0.820, respectively, exceeding the corresponding results of either module used alone. However, the combined improvements were smaller than the arithmetic sum of their individual gains, indicating complementary but non-additive effects. After GhostConv was further introduced, PineRipeSeg achieved corresponding values of 0.981, 0.880, 0.980, and 0.822, while computational cost decreased from 9.6 to 8.7 GFLOPs, model file size decreased from 6.0 to 5.1 MB, and inference speed increased from 103 to 121 FPS. These results indicate that TEDA primarily strengthens fruit–leaf feature discrimination, Inner-IoU refines localization at stricter IoU thresholds, and GhostConv mainly improves computational efficiency. Their combination therefore enables PineRipeSeg to achieve a favorable balance between segmentation performance and computational efficiency.

### 3.4 Comparison of attention mechanisms

The performance of TEDA in field pineapple instance segmentation was compared with that of three commonly used attention mechanisms: SE (Hu et al., 2018), CA (Hou et al., 2021), and CBAM (Woo et al., 2018). For comparability, SE, CA, CBAM, and TEDA were placed at the same network position. To account for stochastic variation during model training, each configuration, including the YOLO11n-seg baseline, was independently trained five times using random seeds of 0, 1, 2, 3, and 4. The dataset split, training parameters, evaluation procedure, and experimental environment were kept unchanged across all runs. The results are reported as the mean ± standard deviation in **Table 6**. Among the general-purpose attention mechanisms, CBAM achieved the highest mean mAP50 values and showed the smallest performance gap relative to TEDA. Therefore, TEDA and CBAM were further subjected to paired statistical comparison to determine whether the observed differences exceeded run-to-run variation.

**Table 6:** Performance comparison among attention mechanisms based on five independent training runs. Values are reported as the mean ± standard deviation.

Model	Box P	Box R	Box mAP50	Mask P	Mask R	Mask mAP50
YOLO11n-seg	0.906 ± 0.006	0.897 ± 0.006	0.931 ± 0.003	0.905 ± 0.007	0.897 ± 0.006	0.928 ± 0.003
YOLO11n-seg+SE	0.909 ± 0.005	0.898 ± 0.006	0.933 ± 0.003	0.908 ± 0.006	0.899 ± 0.006	0.933 ± 0.003
YOLO11n-seg+CA	0.915 ± 0.006	0.908 ± 0.007	0.942 ± 0.003	0.914 ± 0.006	0.907 ± 0.006	0.940 ± 0.002
YOLO11n-seg+CBAM	0.935 ± 0.005	0.916 ± 0.005	0.960 ± 0.001	0.936 ± 0.007	0.916 ± 0.005	0.957 ± 0.001
YOLO11n-seg+TEDA	0.946 ± 0.005	0.925 ± 0.005	0.966 ± 0.001	0.947 ± 0.005	0.925 ± 0.004	0.966 ± 0.002

As shown in **Table 6**, the evaluated attention mechanisms improved the baseline performance to different degrees. The relatively small standard deviations, ranging from 0.001 to 0.003 for mAP50 and from 0.004 to 0.007 for precision and recall, indicate that the results were generally stable across the five independent runs. SE produced only limited improvements because it primarily recalibrates channel weights without explicitly modeling spatial position. CA incorporated directional positional information and achieved higher localization and segmentation performance, while CBAM further improved the results by combining channel and spatial attention.

TEDA achieved the highest mean value for every reported metric. Relative to the baseline, it increased the mean Box mAP50 and Mask mAP50 by 3.5 and 3.8 percentage points, respectively. Compared with CBAM, the strongest general-purpose attention mechanism in this comparison, TEDA increased the mean Box mAP50 and Mask mAP50 by 0.6 and 0.9 percentage points, respectively. Although these absolute differences were modest, the two-tailed paired t-tests across the five runs matched by random seed showed statistically significant differences for both metrics. For Box mAP50, t = 6.325, df = 4, and p = 0.0032; for Mask mAP50, t = 7.894, df = 4, and p = 0.0014. These results indicate that the higher mean performance of TEDA relative to CBAM was consistently observed across the repeated runs and cannot be attributed solely to the run-to-run variation observed in this experiment.

The performance pattern of TEDA is consistent with its task-specific texture-edge design for field pineapple imagery. Pineapple eyes form a multidirectional interlocking texture, whereas sword-shaped leaves exhibit predominantly elongated linear structures. TEDA uses this structural difference to strengthen spatial responses in fruit-texture regions and reduce interference from leaf occlusion and similarly colored backgrounds. Its residual-scaling branch also retains a direct propagation path for the original features and reduces the risk of excessive suppression of informative signals during attention recalibration. The correspondence between this crop-specific structural prior and the dominant fruit–leaf interference in field images provides a plausible explanation for the higher localization and segmentation performance obtained by TEDA in the present task.

### 3.5 Validation in representative field scenes

To evaluate PineRipeSeg under natural field conditions, YOLO11n-seg and PineRipeSeg were applied to the test set. The test set contained 903 images and 1397 pineapple instances, comprising 457 Unripe, 422 Partially ripe, and 518 Ripe instances. Six representative groups of samples were selected for qualitative visual comparison (**Figure 9**), whereas the quantitative instance-level evaluation was performed on the complete test set and is reported in **Table 7**.

**Figure 9:**
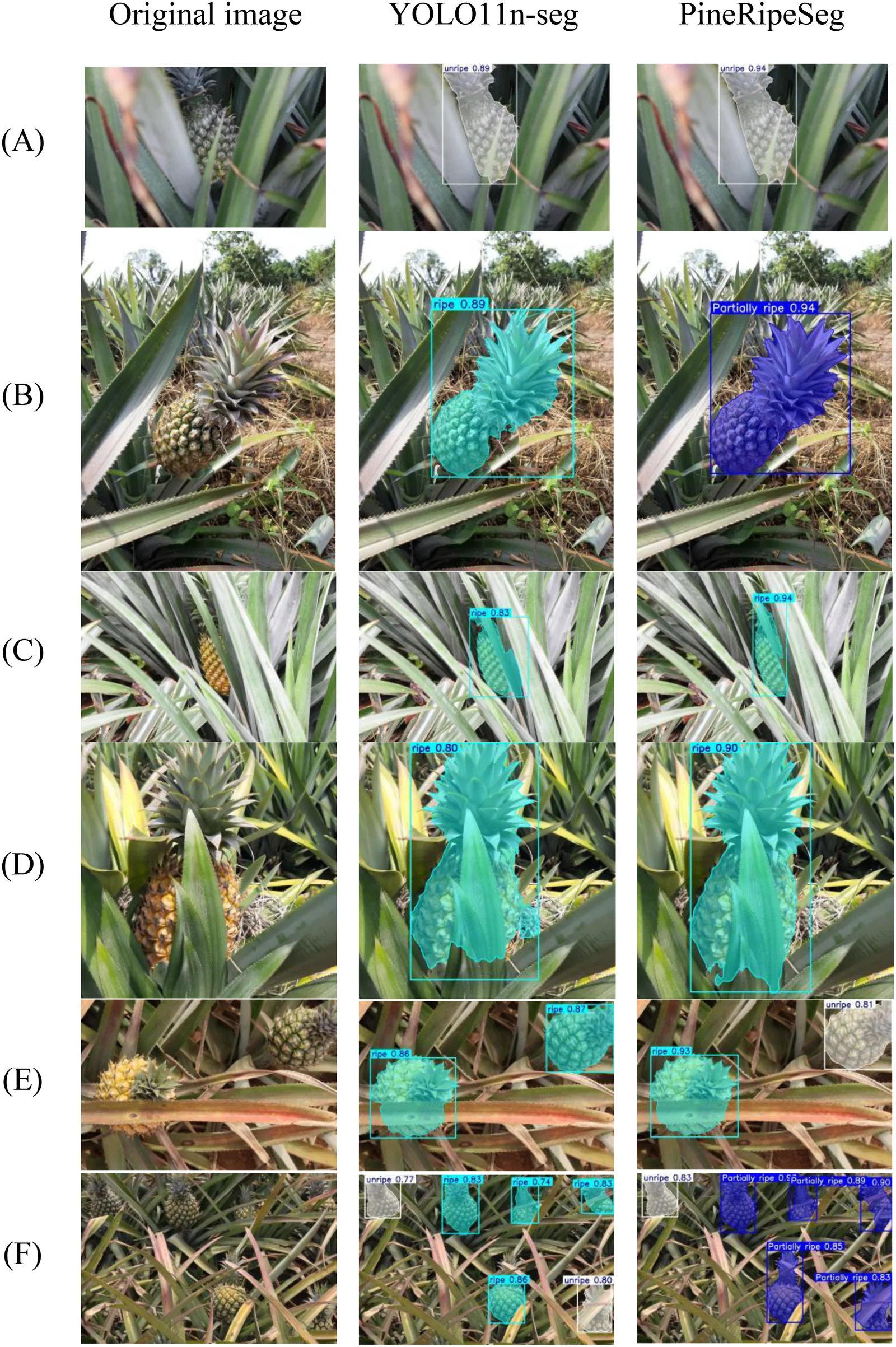
Comparison of model predictions in challenging field scenes. **(A)** Unripe pineapple. **(B)** Partially ripe pineapple. **(C, D)** Ripe pineapples occluded by leaves. **(E, F)** Multi-target scenes.

**Table 7:** Pineapple-instance detection results on the test set.

Method	Detected instances	Predicted unripe	Predicted partially ripe	Predicted ripe	Missed detections	Miss rate (%)
Manual annotations	1397	457	422	518	—	—
YOLO11n-seg	1343	447	335	561	54	3.87%
PineRipeSeg	1385	451	418	516	12	0.86%

**Figures 9A, B** show Unripe and Partially ripe pineapple samples, respectively. Both models correctly recognized the Unripe target, but the mask generated by PineRipeSeg more closely followed the visible fruit boundary and had a higher confidence score. For the Partially ripe target, YOLO11n-seg misclassified the fruit as Ripe and produced a local indentation in the mask at the occluded position. PineRipeSeg correctly classified its ripeness and obtained a more complete instance contour. The result indicates that strengthening pineapple-eye texture and local color-transition features with TEDA reduced reliance on global color information and improved discrimination between adjacent ripeness classes.

It should be noted that the ground-truth masks were generated using the occlusion-aware hybrid annotation protocol described in Section 2.4. Therefore, when a narrow foreground leaf occluded a fruit whose hidden contour could be reliably inferred, the target mask was allowed to extend across the occluded region. In such cases, a predicted mask covering the occluding leaf region does not necessarily indicate segmentation leakage, but may represent agreement with the predefined partially amodal annotation target.

**Figures 9C, D** show ripe pineapples occluded by leaves. YOLO11n-seg correctly classified the targets but included portions of leaves or dry weeds within the segmented fruit regions. Under severe occlusion, its prediction confidence was only 0.80. PineRipeSeg suppressed interference from linear leaf edges and weeds more effectively, produced more accurate contours of the visible fruit surface, and increased the prediction confidence to 0.90. TEDA improved target representation by exploiting the difference between the interlocking texture of pineapple eyes and the linear structure of leaves. Inner-IoU improved bounding-box regression under occlusion and thereby reduced mask overflow beyond the fruit boundary.

**Figures 9E, F** show multi-target scenes. In **Figure 9E**, YOLO11n-seg misclassified the Unripe pineapple on the right as Ripe, whereas PineRipeSeg correctly distinguished the different ripeness classes within the same field of view. **Figure 9F** contains five Partially ripe pineapples and one Unripe pineapple, most of which are visibly occluded. The baseline model misclassified five targets, whereas PineRipeSeg correctly recognized all targets. These results indicate that the improved model represented local texture and color-transition features more consistently when targets were small, occluded, and present alongside multiple ripeness classes.

As shown in **Table 7**, PineRipeSeg detected 42 more pineapple instances than YOLO11n-seg. The number of missed detections decreased from 54 to 12, the miss rate decreased by 3.01 percentage points, and the number of missed detections decreased by 77.8%. Most recognition errors from the baseline model involved the Partially ripe class, whereas the PineRipeSeg predictions for all three classes were closer to the manually annotated counts. Together with the visual results in **Figure 9**, these findings indicate that, within the conditions represented in the current test set, the improved model reduced missed detections and misclassifications in occluded, multi-target, and mixed-ripeness scenes, thereby improving the reliability of pineapple instance segmentation and ripeness classification.

Despite the overall improvement, PineRipeSeg still missed 12 of the 1397 annotated instances in the test set. These residual misses indicate that performance can deteriorate when the visible fruit region is extremely small or divided into several discontinuous fragments by leaves, because the remaining contour, pineapple-eye texture, and color-transition cues may be insufficient to form a stable instance. The validation-set confusion matrix in **Figure 8D** shows that classification confusion occurred mainly around the Partially ripe class, while a small number of background-related errors remained. This pattern is consistent with the continuous appearance transition between adjacent ripeness stages and with field regions in which shadows or senescent leaves resemble parts of the fruit surface. The heatmaps in **Figure 10** show that PineRipeSeg reduced these background responses compared with YOLO11n-seg, but simultaneous color similarity, weak texture contrast, and severe occlusion can still reduce prediction reliability.

**Figure 10:**
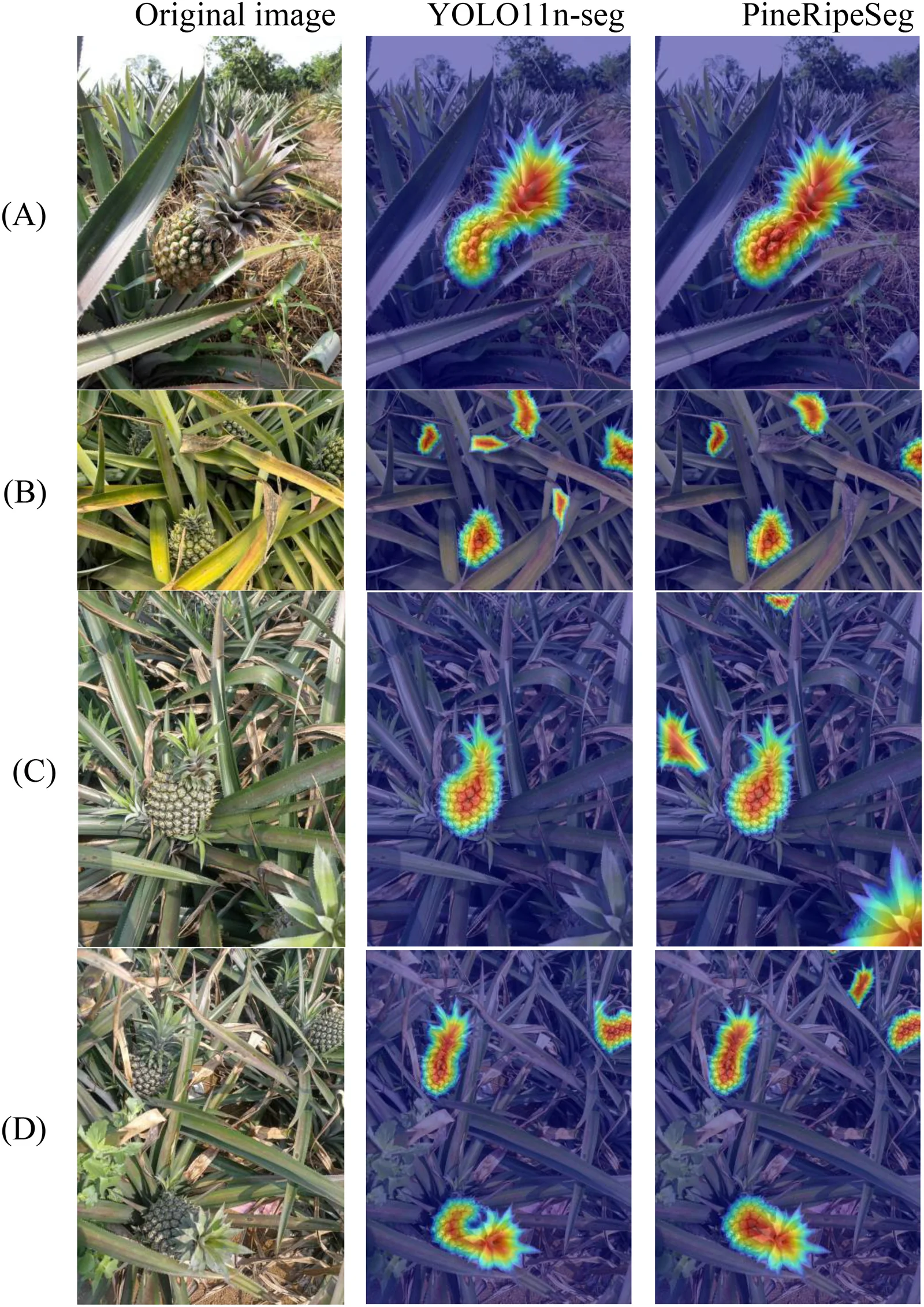
Class-activation heatmaps of model predictions. **(A)** Partially ripe pineapple. **(B)** Senescent leaves with colors similar to ripe fruit. **(C, D)** Severely occluded multi-target scenes.

### 3.6 Heatmap visualization

To qualitatively examine the spatial response patterns associated with model predictions, YOLO11n-seg and PineRipeSeg were visualized using class-activation heatmaps (Selvaraju et al., 2020) (**Figure 10**). Red regions indicate stronger model responses, whereas blue regions indicate weaker responses. These heatmaps are used as qualitative interpretive visualizations and are not intended as quantitative localization or causal explainability measures.

**Figure 10A** shows a Partially ripe pineapple. The strongest responses of YOLO11n-seg were concentrated mainly on the crown, with relatively little response on the fruit surface, and the target was misclassified as Ripe. In contrast, PineRipeSeg showed broader activation over the fruit surface, including regions containing visible pineapple-eye texture and peel-color transitions. This qualitative response pattern was more closely aligned with the fruit-surface regions used for appearance-based ripeness assessment.

**Figure 10B** contains senescent leaves with colors similar to that of ripe fruit. YOLO11n-seg produced strong responses on some yellowed leaves and incorrectly recognized them as pineapples. This response indicates a strong reliance on color and limited discrimination between the interlocking texture of the fruit surface and the linear edges of leaves. PineRipeSeg suppressed irrelevant responses in senescent leaf regions and concentrated its strongest responses on the pineapple surface, indicating improved representation of pineapple-eye texture.

**Figures 10C, D** show severely occluded multi-target scenes. The responses of YOLO11n-seg spread readily to surrounding leaves and the background, while some important target regions showed weak or absent responses, consistent with missed detections. PineRipeSeg focused more consistently on the visible and central regions of each pineapple and retained clearer target responses under occlusion and in multi-target scenes. These results indicate that PineRipeSeg reduced interference from background leaves and similarly colored regions, localized informative features more accurately, and provided a more reliable feature basis for instance segmentation and ripeness classification in complex field environments.

## 4 Discussion

### 4.1 Comparison with existing studies and interpretation of the results

Previous studies on field pineapple vision have generally focused on fruit detection, ripeness recognition, distance estimation, or contour extraction as separate tasks. Chang et al. (2022) combined pineapple maturity recognition with distance estimation, while Lai et al. (2023); Meng et al. (2023), and Zhang R. et al. (2024) improved bounding-box detection under field backgrounds, occlusion, or lightweight-computing requirements. These methods provide reliable fruit locations and, in some cases, ripeness classes, but the rectangular detection region inevitably contains surrounding leaves, crowns, and other background pixels. Shan et al. (2025) used instance segmentation to obtain pineapple positions and geometric contours, although ripeness classification was not performed simultaneously. PineRipeSeg differs from these studies by jointly providing an occlusion-aware hybrid instance mask and an appearance-based ripeness class for each fruit. A related combination of ripeness recognition and instance segmentation has been reported for tomatoes (Wei et al., 2025), but field pineapples present a different feature conflict because the multidirectional texture of pineapple eyes is frequently mixed with the long linear edges of sword-shaped leaves and crowns.

Under the unified dataset, training configuration, and evaluation protocol used in this study, PineRipeSeg obtained higher mask-segmentation accuracy than Mask R-CNN, Mask2Former, and the evaluated YOLO segmentation models, while maintaining a relatively low computational cost. Direct numerical comparison with results reported in other publications remains inappropriate because the datasets, cultivars, annotation principles, image-acquisition conditions, and evaluation protocols differ. The main contribution of PineRipeSeg is therefore not simply a higher metric value, but the lightweight integration of occlusion-aware hybrid instance segmentation and appearance-based ripeness classification for field pineapples under leaf occlusion.

Previous fruit-segmentation studies have shown that edge-guided features can improve contour extraction in complex environments (Sheng et al., 2023). In pineapple images, however, generic edge enhancement may also strengthen the prominent edges of leaves and crowns, which can be more continuous than the fragmented boundary of an occluded fruit. TEDA addresses this conflict by emphasizing the interlocking texture of pineapple eyes while reducing responses to elongated linear structures. The repeated experiments with different attention mechanisms, together with the ablation results and heatmap visualizations, showed that TEDA produced more effective fruit-surface representations and reduced interference from surrounding leaves. GhostConv and Inner-IoU provided complementary contributions by reducing redundant computation and improving the localization of partially visible targets, respectively.

PineRipeSeg achieved a high Mask mAP50, whereas its Mask mAP50–95 remained lower. This gap indicates that detecting a pineapple and obtaining an approximately correct mask are less difficult than accurately fitting a fragmented visible boundary at stricter IoU thresholds. Under severe leaf occlusion, the exposed fruit surface may be divided into small or discontinuous regions. Small deviations in the predicted boundary then have a greater influence at high IoU thresholds and may also contribute to the remaining missed detections.

Uneven natural illumination creates an additional source of performance degradation. Strong shadows may suppress yellow or orange-yellow color transitions and weaken the local contrast of pineapple-eye texture, whereas local highlights may flatten surface detail. These effects are particularly relevant to the Partially ripe class because its appearance lies between the two adjacent ripeness stages, which is consistent with the confusion pattern in **Figure 8D**. Background-related errors may also occur when senescent leaves exhibit both similar color and locally irregular texture. Although TEDA reduces responses to elongated leaf structures, it cannot completely resolve cases in which color, texture, and visible-boundary cues become ambiguous simultaneously.

The environmental factors in the present dataset were naturally distributed rather than predefined as discrete quantitative strata. Although the overall ripeness-class distribution is reported, the current evaluation does not provide condition-specific statistics for different occlusion levels, illumination intensities, or fruit sizes. The present results should therefore be interpreted as an evaluation across the mixed field conditions represented in the dataset rather than as a stratified robustness assessment for individual challenge levels. Future work should construct test subsets stratified by illumination intensity, occlusion ratio, and visible fruit area, and report both class distributions and model performance within each stratum to quantify these effects separately and further improve high-IoU mask quality under extreme field conditions. Further improvements in fine-grained contour accuracy may focus on preserving higher-resolution boundary features and introducing explicit boundary-aware constraints for severely occluded fruit.

### 4.2 Practical meaning and application scope of appearance-based ripeness classification

The ripeness classes used in this study were established according to the appearance-based grading descriptions in NY/T 450-2001. The expansion of yellow or orange-yellow areas over the visible fruit surface was used as an auxiliary quantitative criterion, and the original four stages were consolidated into the Unripe, Partially ripe, and Ripe classes described in Section 2.3. The model output therefore corresponds to an established field-visible grading basis rather than an arbitrary color classification, consistent with the use of external visual traits in fruit maturity detection (Ma et al., 2024).

Pineapple ripening is nevertheless a continuous process. The Partially ripe class covers the transition from predominantly green fruit to fruit with an increasing yellowed area, and its visual boundary with the adjacent classes can be affected by natural illumination, leaf shadows, occlusion, surface damage, and variation among individual fruit. This continuity is also reflected in **Figure 8D**, where most classification errors occurred between Partially ripe and the two adjacent ripeness classes, whereas direct confusion between Unripe and Ripe was limited.

The appearance-based criteria used here should be distinguished from internal physiological maturity and eating quality. NY/T 450–2001 itself notes that fruit-surface color does not always fully reflect internal maturity and that flesh translucency provides a more reliable internal assessment. RGB images can record peel color, texture, and visible morphology, but they do not directly measure soluble solids content, acidity, firmness, or internal translucency. Visible and near-infrared spectroscopy has therefore been investigated for nondestructive assessment of pineapple internal maturity and soluble solids content (Qiu et al., 2023; Semyalo et al., 2024). PineRipeSeg should accordingly be interpreted as a method for identifying externally visible ripeness stages. In a harvesting context, the model therefore provides an external visual ripeness cue rather than a direct measurement of internal fruit quality. Future work may combine RGB appearance information with spectral or physicochemical measurements to establish a more complete relationship between external phenotype and internal quality.

The joint output of ripeness classes and occlusion-aware hybrid instance masks provides more detailed visual information than bounding boxes alone. Under the adopted annotation protocol, each mask represents the annotated fruit extent by retaining visible-region contours and allowing limited local completion only when the occluded contour can be inferred reliably. These outputs may be combined with depth sensing, multiview reconstruction, spatial localization, or picking-point analysis in subsequent system development. Their contribution within an integrated harvesting system should be evaluated together with the other perception, localization, planning, and execution components (He F. et al., 2024; Rajendran et al., 2024; Xiao et al., 2025).

The present dataset contains Bali pineapples collected at two locations and during two acquisition periods. Grouping the images by plot and year reduced the risk of highly related samples appearing in different subsets, but the resulting model still reflects the cultivars, production conditions, and imaging systems represented in the dataset. Further evaluation across additional cultivars, production regions, seasons, and field-management conditions would provide a broader assessment of model generalization.

## 5 Conclusions

To address the difficulty of simultaneously segmenting pineapple instances under occlusion and classifying appearance-based ripeness under severe leaf occlusion and fruit–leaf feature confusion, this study constructed a field pineapple instance-segmentation dataset and developed PineRipeSeg. The main conclusions are as follows:

1. A field dataset containing 6019 images and 9393 Bali pineapple instances was constructed. The fruits were categorized as Unripe, Partially ripe, and Ripe according to the appearance-based grading criteria of NY/T 450-2001, and pineapple instances were annotated using an occlusion-aware hybrid polygon protocol that retained visible regions under uncertain occlusion and allowed limited local contour completion when the hidden boundary could be inferred reliably. The grouping of images by acquisition year and plot reduced the risk of information leakage and provided a reliable basis for evaluating model performance under field-domain variation.
2. PineRipeSeg was developed based on YOLO11n-seg. Its core component is the proposed Texture-Edge Decoupling Attention module, which uses the structural difference between the multidirectional interlocking texture of pineapple eyes and the linear edges of leaves and crowns to enhance visible fruit-surface features and suppress non-fruit interference. GhostConv and Inner-IoU were incorporated to reduce redundant computation and improve the localization of occluded fruit, respectively. The coordinated design enabled PineRipeSeg to jointly perform occlusion-aware hybrid instance segmentation and appearance-based ripeness classification while retaining a lightweight network structure.
3. PineRipeSeg achieved Box mAP50, Mask mAP50, and Mask mAP50–95 values of 0.981, 0.980, and 0.822, respectively, with a computational cost of 8.7 GFLOPs. The model comparisons, ablation experiments, repeated attention-mechanism evaluations, challenging-scene results, and heatmap visualizations consistently showed improved representation of visible pineapple surfaces and reduced interference from leaves and similarly colored background regions. These results demonstrate that PineRipeSeg provides an effective lightweight solution for pineapple instance segmentation and appearance-based ripeness classification in natural field environments. The present evaluation was conducted on Bali pineapples collected at two field locations in Guangdong Province, providing a defined experimental basis for subsequent evaluation across broader production conditions.

## Data Availability

The original contributions presented in the study are included in the article/supplementary material, further inquiries can be directed to the corresponding author/s.
